# A Single-Blind randomized controlled trial to evaluate the effect of extended counseling on uptake of pre-antiretroviral care in eastern uganda

**DOI:** 10.1186/1745-6215-12-184

**Published:** 2011-07-27

**Authors:** Lubega Muhamadi, Nazarius M Tumwesigye, Daniel Kadobera, Gaetano Marrone, Fred Wabwire-Mangen, George Pariyo, Stefan Peterson, Anna Mia Ekström

**Affiliations:** 1 District Health Office, Iganga District Administration, PO Box 358, Iganga, Uganda; 2 Department of Epidemiology and Biostatistics, Makerere University School of Public Health, PO Box 7072, Kampala, Uganda; 3 Makerere University Iganga/Mayuge Health & Demographic Surveillance System PO BOX 7072 Kampala Uganda; 4 Division of Global Health, IHCAR, Department of Public Health Sciences Karolinska Institutet, Sweden; 5 Department of Health Policy Planning and Management, Makerere University School of Public Health, PO Box 7072, Kampala, Uganda; 6 IMCH, Department of Women's and Children's Health, Uppsala University, Sweden; 7 Institute of Health Sciences Busoga University, PO Box 154, Iganga, Uganda; 8 Department of Infectious Diseases, Karolinska University Hospital, Sweden

## Abstract

**Background:**

Many newly screened people living with HIV (PLHIV) in Sub-Saharan Africa do not understand the importance of regular pre-antiretroviral (ARV) care because most of them have been counseled by staff who lack basic counseling skills. This results in low uptake of pre-ARV care and late treatment initiation in resource-poor settings. The effect of providing post-test counseling by staff equipped with basic counseling skills, combined with home visits by community support agents on uptake of pre-ARV care for newly diagnosed PLHIV was evaluated through a randomized intervention trial in Uganda.

**Methods:**

An intervention trial was performed consisting of post-test counseling by trained counselors, combined with monthly home visits by community support agents for continued counseling to newly screened PLHIV in Iganga district, Uganda between July 2009 and June 2010, Participants (N = 400) from three public recruitment centres were randomized to receive either the intervention, or the standard care (the existing post-test counseling by ARV clinic staff who lack basic training in counseling skills), the control arm. The outcome measure was the proportion of newly screened and counseled PLHIV in either arm who had been to their nearest health center for clinical check-up in the subsequent three months +2 months. Treatment was randomly assigned using computer-generated random numbers. The statistical significance of differences between the two study arms was assessed using chi-square and t-tests for categorical and quantitative data respectively. Risk ratios and 95% confidence intervals were used to assess the effect of the intervention.

**Results:**

Participants in the intervention arm were 80% more likely to accept (take up) pre-ARV care compared to those in the control arm (RR 1.8, 95% CI 1.4-2.1). No adverse events were reported.

**Conclusions:**

Provision of post-test counseling by staff trained in basic counseling skills, combined with home visits by community support agents had a significant effect on uptake of pre-ARV care and appears to be a cost-effective way to increase the prerequisites for timely ARV initiation.

**Trial registration:**

The trial was registered by Current Controlled Trials Ltd C/OBioMed Central Ltd as ISRCTN94133652 and received financial support from Sida and logistical support from the European Commission.

## Background

Post-test counseling is a vital tool for uptake and adherence to regular pre-antiretroviral (pre-ARV) care and timely entry into antiretroviral care (ART) for people living with HIV (PLHIV) [1-4]. The counseling also helps PLHIV fight stigma, disclose their HIV status and adhere to ART when initiated [2,5-8]. The WHO provides post-test counseling guidelines for PLHIV [4], but in many low-income settings these guidelines are not followed, partly because the staff are overloaded and/or have not been trained in basic counseling skills [9]. Consequently, many newly screened PLHIV in such settings do not appreciate the importance of regular pre-ARV care resulting into low uptake of pre-ARV or high loss to follow up during pre-ARV care [10-16].

In Iganga district, Uganda, over 90% of the 1400 newly screened PLHIV between 2004 and 2007 never attended pre-ARV care or were lost to follow-up during pre-ARV care. In Iganga district, as commonly encountered throughout Sub-Saharan Africa, post-test counseling at the ART clinic is conducted by staff who lack basic training in counseling skills. Staff are often overloaded as they also have been assigned work in other departments in addition to their tasks in the ART clinic (task piling). Most normally neither have access to, nor follow the WHO counseling guidelines when offering HIV counseling services (District health office 2009-unpublished).

In December 2008, our research team conducted a qualitative study to explore reasons for failure to take up or loss to follow up of PLHIV during pre-ARV care in eastern Uganda interviewing PLHIV, half of whom had dropped out of pre-ARV care, and their next of kin. The recurrent theme for failure to take up or adherence to regular pre-ARV care was inadequate post-test counseling for PLHIV on when and why they should return for care [17].

A randomized controlled trial was thus conducted to assess whether a low-cost intervention through providing post-test counseling by staff with training in basic counseling skills, combined with home visits by community support agents for extended counseling, could improve uptake of pre-ARV care among newly diagnosed PLHIV, minimize loss to follow-up during pre-ARV care, and, subsequently enhance timely initiation of ART in this resource-poor district in eastern Uganda.

## Methods

### Trial design

This was a parallel group balanced (1:1) client randomized and single blind superiority controlled trial conducted in three health facilities in Iganga district, eastern Uganda.

### Eligibility criteria

Eligible participants consisted of all newly screened HIV-positive adult clients (>18 yrs) at the three recruitment centres at the start of the study, who were of sound mental status, and who were not under the prevention of mother to child transmission (PMTCT) program or bound to leave the district during the period of follow-up.

### Study setting and location

The study was conducted in Iganga district located approximately 115 km east of the capital Kampala between July 2009 and June 2010. The three recruitment centres included Iganga district hospital and two Health Centre (HC) IVs of Kiyunga and Busesa Health Sub Districts (HSD). These centres were purposively selected because they had a constant supply of test kits and active pre-ARV clinics. The hospital serves a catchment area of approximately 600,000 people while each of the two HC IVs serves a population of approximately 100,000 people. There are 101 health units in the district, 70 of which are HC II, 27 HC III, 3 HC IV and one general hospital.

Ideally all health units should offer voluntary counselling and testing (VCT) and other aspects of pre-ARV care, but currently only the HC at level IV and the hospital actually offer these services. The lower level units (HC II and III) normally run out of stock of HIV test kits and cotrimoxazole (District health office 2009-unpublished). The district has over 200 private drug shops/private clinics scattered in the main Iganga town and many others in small towns in the district. These drug shops/private clinics are not officially accredited to offer pre-ARV care but often sell cotrimoxazole which is one important component of pre-ARV care to PLHIV (District health office 2009-unpublished).

Over 90% of the residents in the district are rural subsistence farmers and the majority belong to the Basoga tribe (District health office 2009-unpublished).

Approximately 40,000 (6.7% of the district adult population) are estimated to be living with HIV with over 6,000 of PLHIV presumed to be eligible for ART (MOH, 2006, UNAIDS/WHO, 2007). Currently 3500 PLHIV regularly attend pre-ARV care and approximately a 1000 are on ART in the district [18,19].

VCT is conducted either on client's demand or occasionally when demanded by the clinicians on suspicion of HIV infection or AIDS. The post-test counseling is however mostly conducted by nurses or nursing assistants attached to the ART clinic.

### Description of the intervention

The design involved following newly screened PLHIV after post-test counseling for uptake of pre-ARV care at any HC (level II-V) within the district which was nearest to their homes. Following a positive HIV test, the participants were randomized into one of two arms: the control arm and the intervention arm. The participants in the control arm received standard care including post-test counseling by staff not trained in basic counseling skills at any of the three recruitment centres. Standard counseling included assignment of an identification number, declaration of the results, provision of cotrimoxazole prophylaxis and advice to the client to go for pre-ARV care at the nearest HC every three months.

The participants in the intervention arm received another form of counseling and follow-up. The specialized counseling was conducted by six health workers who had been trained on basic counseling skills for three days at a direct financial cost of twenty (20) USD per staff. During the training, it was emphasized that those who had been trained should not share their new skills with the staff selected for the control arm. The counseling offered in the intervention arm similarly involved allocation of an identification number, declaration of the results to the client and cotrimoxazole prophylaxis. In addition, the counsellor encouraged self-disclosure of HIV status to the participant's immediate family, promoted positive living with HIV, HIV prevention and emphasized the importance of going for pre-ARV care at the nearest HC every three months. As part of the intervention, these participants were also attached to an HIV/AIDS community support agent close to their area of residence who visited them monthly at their homes for a two hour counseling session and reminded them to go to the nearest health centre for quarterly pre-ARV care. HIV/AIDS community support agents are influential volunteers in the community or ART expert clients who have been sensitized and assigned the role of counseling and encouraging PLHIV to seek care and subsequently link them to the service providers. The community support agents had been trained and registered by the district in previous five years but they also received a one-day orientation about the present study.

### Outcome measures

**Figure 1:**
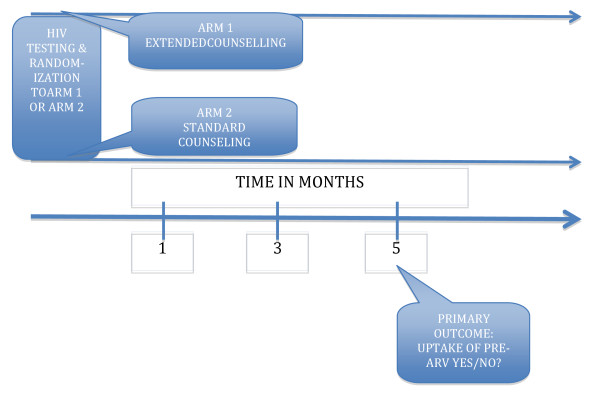
**Description of the trial time line for assessing uptake of pre-ARV care in Iganga district**.

The outcome measure was the proportion of newly detected and counseled PLHIV in either arm who had been to their nearest HC for clinical check-up in the subsequent three months after enrollment. An allowance of extra two months to return for check-up was considered for every participant to cater for any inevitable circumstances that would prohibit the participant from going for check-up. The participants in both arms were followed up and considered to have taken up (accepted) or not taken up (not accepted) pre-ARV care according to the following definition: those who went for clinical check-up at the nearest HC at least once in the subsequent three months (+ 2 months, see above) were considered to have taken up pre-ARV care, while those who missed their scheduled appointment to the pre-ARV clinic in the subsequent three months (+2 months) were considered not to have taken up pre-ARV care (Figure 1).

### Independent variables

Information was collected on the following important factors or potential confounders to pre-ARV uptake; socio-demographic characteristics such as age, sex, education, religion, marital status, marriage status, occupation and number of people in the house-hold. Information was also collected on knowledge of HIV transmission, prevention, pre-ARV care and its importance and stigma. For participants who went to their nearest HC for pre-ARV care after 3 months (+ 2 months) information on reasons for going for care, disclosure and motivational factors for disclosure, any side effects of cotrimoxazole, constraints to pre-ARV care seeking, condom use and number of sexual partners was also collected.

### Data collection techniques

The baseline data for every enrolled participant was collected through face-to face interviews following a short structured questionnaire by a trained research assistant at each of the respective three recruitment centres. Every enrolled participant was then scheduled to seek pre-ARV care after 3 months at their nearest HC jointly agreed on by the counselling staff and the participant. A research assistant identified at each of these agreed health centres collected data from the study participants at the end of the follow-up period.

All the counsellors and corresponding research assistants were trained for three days on the study aim, design and tools. The study tools were pre-tested using a pilot study at another HC IV offering ART in the area. Experiences from the pilot study were discussed at an extra session separately for each arm. Necessary changes were then made to the tools and the research assistants were given additional guidance. Throughout the study period, random validity checks at the HC were conducted by the first and second author (LM and TN) to ensure compliance, and also to collect the completed corresponding questionnaires. The completed questionnaires were then checked for consistency, range of values and completeness.

### Sample size

The sample size was calculated using a formula suggested by both Altman and Bland [20,21]. At a power of 80%, estimated pre-ARV uptake of 50% for the control arm, a target to detect a difference of absolute 14% in the intervention arm as significant at 5% level, a minimum of 193 participants (rounded up to 200) per arm was found adequate. The proportion of newly screened PLHIV normally differs between the different study centres. I It is twice as high at Iganga hospital compared to Busesa and Kiyunga respectively. Based on these proportions, 50% (200 participants 100 in each arm) were enrolled at Iganga hospital and 25% (100 participants 50 in each arm) were recruited at each of the other two recruitment centres (District health office 2009-unpublished).

### Randomization and masking

In all the study centres, participants were randomly assigned to receive the standard post-test counseling or extended counseling. Treatment assignment was randomly generated in blocks of four based on computer generated random number sequences provided by one of the statisticians in the study team (DK) who had no contact with the study participants. Assignment sequences were placed in opaque sealed envelopes and appropriate numbers sent to each of the study centres. At each centre, participant enrollment was done by an assigned laboratory technician who carried out the HIV testing. After a client had tested HIV positive, the technician at each centre asked the eligible client to pick and open an envelope from a batch of four for assignment to the appropriate arm. At each centre this procedure continued until the required allocated numbers per arm had been realized. Given the nature of the intervention, it was hard to mask the staff in the different arms as to which arm they themselves belonged. None of the study subjects, however, was informed of the details of the arm to which they had been assigned. To limit contamination between and among the research assistants and the participants, however, each arm at every centre was put in different rooms at least 50 metres apart. The staff members were also instructed not to share the nature and content of counselling they offered to the participants.

### Data management and analysis

All the completed and edited questionnaires were double-entered and cleaned using using Epi-Info version 2000. The cleaned data were then exported to STATA8 (STATA Corporation, college station TX USA) where analysis was carried out in three stages.

Stage 1 involved a description of baseline characteristics of the participants in the two arms to find out how comparable they were with respect to potential confounders such as age, education, and marital status. Chi-square and the t-test were used to test for any significant differences between the two arms for categorical and quantitative data respectively.

Stage 2 involved bivariate analyses to establish the relationship between the different independent variables and the outcome variable. The risk ratios (RR) comparing uptake of pre-ARV care in intervention arm to the control arm, were computed as well as their 95% confidence intervals. All variables where the association with the outcome variable had a p-value less than 0.2 were considered important for inclusion in the next stage of analysis. A stratified analysis in which the computed risk ratios for different levels of each independent variable were pooled using the Mantel-Haenszel Risk Ratio (MHRR) was also performed. The MHRR was performed to adjust for differences in risk ratios of uptake of pre-ARV at different levels of each independent variable. A Mantel-Haenszel procedure is robust and offers an elementary alternative of computing a common rate ratio to the earlier used maximum likelihood estimates[22].

Stage 3 was a multivariable analysis where different models assessing the independent effect of the intervention on uptake (primary outcome) to pre-ARV care were examined by controlling for the different potential confounders. The multivariable analysis started by computing the RR generated by the intervention alone on uptake of pre-ARV care. Other models were then computed by cumulatively adding each of the independent variables that had been significant in stage two above until all the variables had been exhausted. The intervention was considered significant if the (RR) did not change significantly for the different generated models.

### Ethical clearance

This study was approved by the Makerere University School of Public Health Institutional Review Board (ref MUSPH 06012009) and the Iganga district authorities. Before enrolment into the study, the respondents were informed about the study aims, their discretion to participate or withdraw at any time and assured that all information obtained from them would be kept confidential. The anticipated benefits or risks of the study to the participants or the community were clearly explained and all the study participants signed consent forms before the interviews commenced.

## Results

**Figure 2:**
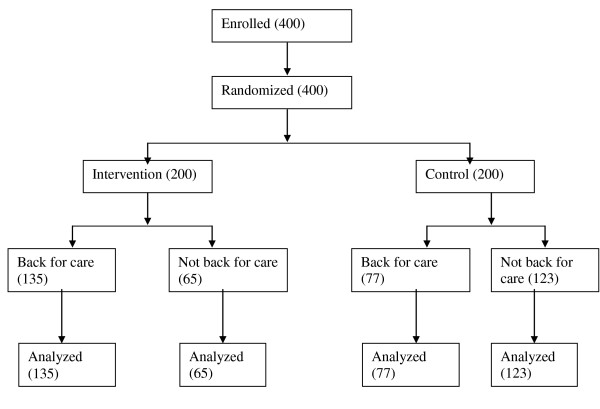
**Consort participant flow diagram from enrolment to analysis**.

The study participant flow included a total of 400 newly screened PLHIV who were deemed eligible and enrolled for the study. The 400 participants were randomly assigned with 200 to the intervention arm and 200 to the control arm. In the intervention arm, 135/200 clients took up and returned for pre-ARV care and 65/200 did not return for pre-ARV care. In the control arm, 77/200 clients took up and returned for pre-ARV care while 123/200 did not return for pre-ARV care (Figure 2).

The majority of the participants in both arms were comparable with regard to gender, (p = 0.75), age (p = 0.48), education status (p = 0.83), marriage status (p = 0.90), occupation (p = 0.62), distance of the PLHIV to the centre (p = 0.56) and number of people living in the household together with PLHIV (p = 0.23). There were, however, observed differences between the two arms with regard to pre-ARV awareness (p = 0.01), religion (p = 0.02) and marital status (p = 0.02) although on further analysis these differences did not significantly influence the effect of the intervention on the outcome (tables 1, 2 and 3) Both the bivariate and multivariable analyses were conducted using intention-to-treat with a sample size of 200 participants for the intervention arm and 200 participants for the control arm. In the bivariate analysis, comparison of the two arms in relation to the outcome showed that participants in the intervention arm were more likely to return for care compared to those in the control arm with an overall percentage of 38.5% and 67.5% for the control and intervention arms respectively and an unadjusted RR of 1.8 (95% CI: 1.4-2.1) (table 2). Thus participants in the intervention arm were 80% more likely to take up pre-ARV care compared to those in the control arm.

**Table 1:** Description of background characteristicsto test if there were any significant differences between the control and intervention arms in Iganga district (N = 400).

Characteristic	Control (n = 200) Number (%)	Intervention (n = 200) Number (%)	Chi-square/t value (P-value)
**Centre**			
Centre 1 (Busesa)	50 (25.0)	50 (25.0)	
Centre 2 (Iganga)	100 (50.0)	100 (50.0)	
Centre 3 (Kiyunga)	50 (25.0)	50 (25.0)	0.000 (1.00)
**Sex**			
Male	73 (36.5)'	70 (35.0)	
Female	127 (63.5)	130 (65.0)	0.098 (0.75)
**Age (years)**			
18-24	39 (19.5)	29 (14.5)	
25-34	57 (28.5)	68 (34.0)	
35-44	58 (29.0)	56 (28.0)	
45-70	46 (23.0)	47 (23.5)	2.480 (0.48)
**Education**			
Low education	130 (65.0)	128 (64.0)	
Well educated	70(35.0)	72 (36.0)	0.044 (0.83)
**Marital status**			
Unmarried	82 (41.0)	61 (30.5)	
Married	118 (59.0)	139 (69.5)	4.799 (0.02)
**Marriage status**			
Monogamous	62 (52.1)	72 (51.8)	
Polygamous	56 (47.9)	67 (48.2)	0.014 (0.90)
**Occupation**			
Farmer	134 (67.0)	140 (70.0)	
Trader/business	48 (24.0)	40 (20.0)	
Salary/wage earner	18 (9.0)	20 (10.0)	0.964 (0.62)
**Distance to unit (mean)**	21.0 (SD = 19.6)	22.0 (SD = 21.1)	0.590 (0.56)
**Household size (mean, SD)**	6.1 (SD = 3.8)	6.7 (SD = 5.6)	1.200 (0.23)
**Religion**			
Christians	117 (58.5)	140 (70.0)	
Muslims	83 (41.5)	60 (30.0)	5.757 (0.02)
**Pre-ARV awareness**			
Aware	56	(28.0)	81 (40.5)
Not aware	144 (72.0)	119 (59.5)	6.940 (0.01)

**Table 2:** A percentage comparison of uptake of pre-ARV care in the control and intervention arm stratified for different categories of the baseline characteristics in Iganga district (reference = control arm)

Variable	Control (N = 200)	Intervention (N = 200)	p-value ratio	Crude risk (95% CI)	(MH risk ratios)
**Recruitment Centre**					
Centre 1 (Busesa)	36.0	68.0	0.003	1.9 (1.3-2.9)	1.8 (1.4-2.1)
Centre 2 (Iganga)	39.0	68.0	<0.001	1.7 (1.3-2.3)	
Centre3 (Kiyunga)	40.0	66.0	0.013	1.7 (1.1-2.5)	
**Sex**					
Male	30.1	64.3	<0.001	2.1 (1.4-3.2)	1.7 (1.4-2.1)
Female	43.3	69.2	<0.001	1.6 (1.3-2.0)	
**Age group**					
18-24	41.0	65.5	0.050	1.5 (1.1-2.5)	1.8 (1.4-2.2)
25-34	35.1	64.7	0.002	1.8 (1.2-2.7)	
35-44	44.8	71.4	0.006	1.6 (1.2-2.2)	
45-70	32.6	68.1	0.002	2.0 (1.3-3.3)	
**Education**					
Low education	40.0	69.5	<0.001	1.7 (1.4-2.2)	1.8 (1.4-2.2)
High education	35.7	63.8	0.001	1.8 (1.3-2.6)	
**Marital status**					
Unmarried	39.0	68.5	<0.001	1.8 (1.3-2.4)	1.8 (1.4-2.1)
Married	38.1	66.9	<0.001	1.8 (1.4-2.3)	
**Marriage status**					
Monogamous	35.5	65.3	0.001	1.8 (1.3-2.7)	1.8 (1.4-2.3)
Polygamous	39.0	68.9	<0.001	1.8 (1.3-2.4)	
**Occupation**					
Farmer	40.3	65.0	<0.001	1.6 (1.3-2.1)	1.8 (1.4-2.1)
Trader	39.6	72.5	0.003	1.8 (1.2-2.7)	
Salary earner	22.2	75.0	0.008	3.4 (1.4-8.3)	
**Religion**					
Christians	43.6	75.0	<0.001	1.7 (1.4-2.2)	1.7 (1.4-2.1)
Muslims	31.3	50.0	0.024	1.6 (1.1-2.4)	
**Pre-ARV awareness**					
Aware	60.7	86.4	0.002	1.4 (1.1-1.8)	1.6 (1.3-2.0)
Not aware	29.9	54.6	<0.001	1.8 (1.1-1.8)	
**Overall analysis**	38.5	67.5	0.001	1.8 (1.4-2.1)	

**Table 3:** Multivariable analysis to assess the effect of the intervention (extended counselling) on uptake of pre-ARV care in Iganga district.

Model/Variable	Risk ratio (95% CI)	P value
**Model 1**		
Intervention*	1.8 (1.4-2.1)	<0.001
**Model 2**		
Intervention*	1.7 (1.4-2.1)	<0.001
Sex (female)	1.1 (1.0-1.4)	
**Model 3**		
Intervention*	1.7 (1.4-2.1)	<0.001
Sex (female)	1.2 (1.0-1.4)	
Education(primary +)	0.9 (0.8-1.1)	
**Model 4**		
Intervention*	1.7 (1.4-2.1)	<0.001
Sex (female)	1.1 (0.9-1.4)	
Education (primary +)	0.9 (0.8-1.1)	
Age group (18-25 yrs)		
25-34	1.0 (0.7-1.2)	
35-44	1.1 (0.8-1.4)	
45-70	1.0 (0.6-1.3)	
**Model 5**		
Intervention*	1.7 (1.4-2.1)	<0.001
Sex (female)	1.2 (0.9-1.4)	
Education (primary +)	0.9 (0.8-1.1)	
Age group (18-25 yrs)		
25-34	0.9 (0.7-1.2)	
35-44	1.1 (0.8-1.4)	
45-70	1.0 (0.8-1.3)	
Centre 2 (Iganga hosp)	1.0 (0.8-1.2)	
Centre 3 (Kiy HSD)	1.0 (0.8-1.2)	
**Model 6**		
Intervention*	1.8 (1.4-2.1)	<0.001
Sex (female)	1.1 (0.9-1.4)	
Education (primary +)	0.9 (0.8-1.1)	
Age group (18-25 yrs)		
25-34	0.9 (0.7-1.2)	
35-44	1.1 (0.8-1.4)	
45-70	1.0 (0.7-1.3)	
Marital status (married)	1.0 (0.8-1.2)	
**Model 7**		
Intervention*	1.8 (1.4-2.1)	<0.001
Sex (female)	1.2 (0.9-1.4)	
Education (primary +)	0.9 (0.8-1.1)	
Marital status (married)	1.0 (0.8-1.2)	
Occupation (trader)	1.1 (0.9-1.3)	
Occupation (salaried)	1.1 (0.8-1.2)	
**Model 8**		
Intervention*	1.8 (1.4-2.1)	<0.001
Sex (female)	1.2 (1.0-1.4)	
Education (primary +)	0.9 (0.8-1.1)	
Marital status (married)	1.00 (0.8-1.2)	
Occupation (trader)	1.1 (0.9-1.3)	
Occupation (salaried)	1.1 (0.8-1.4)	
Centre 2 (iganga hosp)	0.9 (0.8-1.2)	
Centre 3 (Kiy HSD)	0.9 (0.7-1.2)	

*Significant at 5% level

The effect of the intervention remained significant after adjusting for the combined effect of each of the independent variables in stratified analysis with a MHRR of pre-ARV uptake in intervention arm compared to the control arm ranging from 1.6 to 1.8. Specifically, the MHRRs and their 95% CIs shown by the independent variables were as follows: recruitment health centre (Busesa) 1.8 (95% CI 1.4-2.1), sex (male) 1.7 (95% CI 1.4-2.1), age group (18-24 yrs) 1.8 (95% CI 1.4-2.2), education (low education) 1.8 (95% CI 1.4-2.2), marital status (unmarried) 1.8 (95% CI 1.4-2.1), marriage status (monogamous) 1.8 (95% CI 1.4-2.3), occupation (subsistence farmer) 1.8 (95% CI 1.4-2.1), religion (Christians) 1.7 (95% CI 1.4-2.1) and pre-ARV awareness (aware) 1.6 (95% CI 1.3-2.0) (table 2). A test of homogeneity of the combined MHRR showed no significant difference among the strata. The result re-affirms the significant effect of extended counselling on uptake of pre-ARV care.

All models generated in the multivariable analysis to control for potential confounders did not significantly change the adjusted risk ratio or the 95% confidence interval imposed by the intervention alone on the outcome RR 1.8 (1.4-2.1) (table 3)

**Figure 3:**
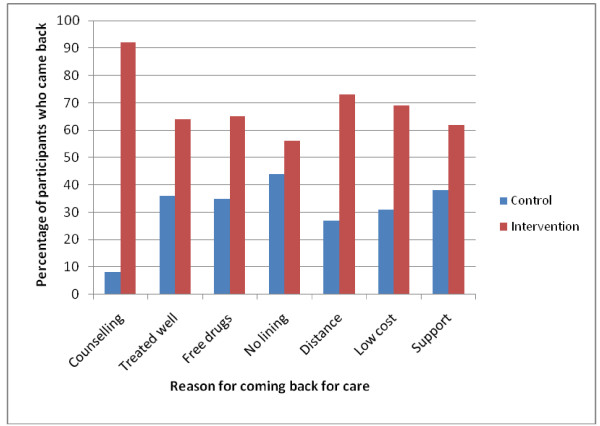
**Participant reasons for coming back for care in both arms in Iganga district**.

All participants in both arms were well informed about HIV transmission, prevention and AIDS defining symptoms. The results also show that counseling was a major reason for participants in the intervention arm to come back for care (91.6%) compared to (8.4%) in the control arm (Figure 3). In addition, the majority of clients in the intervention arm (64.5%) of those who came back for pre-ARV care had disclosed their HIV status to their next of kin compared to (34.5%) in the control arm.

## Discussion

We show that low-cost extended counseling carried out by low-level health staff with basic training in counseling skills complimented with monthly visits to newly screened PLHIV by community support agents, may considerably enhance uptake of pre-ARV care. The study also shows that the majority of clients in the intervention arm had also reportedly disclosed their HIV status to members of the immediate family compared to those in the control arm.

### Trial limitations

All the staff members who conducted the counseling at the different centres were from the same units and had worked together in these units for a long time, thus complete decontamination between staff was hard to ensure but genuine efforts were made during pre-trial training to inform them about the importance of avoiding discussing the intervention.

It was also impossible to entirely ensure that the participants did not share information about the trial between themselves although this is unlikely given the sensitivity of the topic and the high stigma still associated with HIV in the community [23].

Since the intervention did not involve following up of the community support agents, it was also difficult to ascertain the quality and quantity of counseling they provided to the participants during follow up visits in their homes. It is equally hard to ascertain whether it was the initial post-test counseling provided by the staff at the unit in the intervention arm, and/or the follow up counseling provided by the community support agents that encouraged participants to come back for pre-ARV care. Unpackaging the intervention could thus be a subject for further research.

### Interpretation and generalizability

Appropriate randomization of participants was done and the association between the intervention and the outcome variable should not have been affected by participant allocation bias resulting from baseline imbalance (table 1). Participants in the intervention arm were 1.8 times more likely to take-up pre-ARV care compared to those in the control arm regardless of differences in baseline characteristics. The multivariable analysis showed that the risk ratios generated by the intervention remained similar and statistically significant across different models adjusted for confounding. Our interpretation is thus that the high uptake of pre-ARV care in the intervention arm could be explained by the more adequate counseling offered by staff who had trained in basic counseling skills. This finding is also supported by other studies where appropriate counseling has been found to be vital for health seeking behavior of PLHIV [4,24-29].

The HIV/AIDS community support agents that offered further counseling through home visits, encouraging participants to go the nearest health centre for pre-ARV care also acted as primary care linkages between the participants and the health care system. The importance of primary linkages as a tool for improving health seeking behavior and access to comprehensive HIV care has also previously been outlined by other studies in resource-poor settings [30-34].

From a secondary prevention perspective, it is important to note that all participants in both arms were knowledgeable about the modes of HIV transmission, prevention and HIV defining symptoms. This could possibly be attributed to the Uganda government's multi-sectoral open policy response to the HIV epidemic, which, since the 1980s, has increased HIV awareness among the population [13,35-39].

More participants in the intervention arm who came back for care had reportedly disclosed their sero-status to their next of kin compared to participants in the control arm. This could be explained by the adequacy of counseling provided in the intervention arm. The relationship between adequate counseling and HIV status disclosure has also been demonstrated previously [2,8,40-51].

## Conclusions

The trial demonstrates that if newly screened PLHIV receive specialized counseling by staff who have received low-cost training in basic counseling skills, combined with home visits by community support agents, they are up to 80% more likely to take up pre-ARV care. The trial further indicates that extended counseling may also help clients overcome or reprioritize among other barriers to comprehensive HIV care in resource-poor settings such as high transport costs, as well as enhance social support by encouraging status disclosure. Community support agents may supplement the efforts of already overloaded staff at ART clinics and make the health system more efficient. The findings of the trial may therefore form a foundation for low cost measures needed to increase uptake of HIV care and subsequent follow-up of PLHIV as a cornerstone for retention of PLHIV under comprehensive HIV care in resource poor settings.

The cost in terms of the training in basic counseling skills was only 20 US dollars per staff, a sum that should be comparable in other rural contexts in sub-Saharan Africa. In similar settings that are able to mobilize volunteer community support agents, a similar intervention would be feasible. The authorities would, however, need to address other important system deficiencies such as stock-outs of HIV testing kits and cotrimoxazole, poor staff attitudes towards pre-ARV care and staff confidentiality, that are common in similar settings [17,52].

The trial findings could be applicable to other Ugandan rural districts and other areas in sub-Saharan Africa with similar health system and community settings.

## Abbreviations

ART: Antiretroviral therapy; ARV: Antiretroviral drugs; CI: Confidence interval; HC: Health centre; HSD: Health sub district; MHRR: Mantel-haenszel risk ratio; MOH: Ministry of health; PLHIV: People living with HIV/AIDS; PMTCT: Prevention of mother to child transmission; RR: Risk ratio; USD: United states dollars; VCT: Voluntary counseling and testing.

## Competing interests

The authors declare that they have no competing interests.

## Authors' contributions

LM and TN were involved in the inception, design, data collection, analysis, interpretation and manuscript writting for the study. All the authors were substantively involved in the design, analysis, interpretation and manuscript writing for the study.

## Funding

This trial received financial and logistical support from Sida and the European Commission's 6^th ^framework programme, under the ARVMAC project http://www.arvmac.eu.

The report has been compiled according to the CONSORT 2010 updated guidelines for reporting parallel group randomized trials.
